# Evaluation of new variables to predict delirium outcome

**DOI:** 10.1186/cc12651

**Published:** 2013-06-19

**Authors:** JPLM de Carvalho, R Alvim, JCS Martins, C Bouza, P Zenaide, R Zantieff, B Pondé, D Amorim, LC Quarantini, D Gusmao-Flores

**Affiliations:** 1 Faculdade de Medicina da Bahia, Universidade Federal da Bahia, Salvador, BA, Brazil; 2 Escola Bahiana de Medicina e Saúde Pública, Salvador, BA, Brazil; 3 University Hospital Prof. Edgard Santos, Universidade Federal da Bahia, Salvador, BA, Brazil

## Introduction

Delirium assessment is already a well-established practice in the ICU. Usually, these evaluations are represented as delirium incidence or delirium-free days without coma. We propose four derived variables to predict the outcome in delirium patients in order to identify the most accurate data.

## Methods

A prospective study took place in the ICU of the University Hospital Professor Edgard Santos, Salvador, Brazil during the period of January to March 2013. Adult patients were assessed twice daily for detection of delirium with the Confusion Assessment Method for the ICU. Those patients with less than 48 hours in the ICU were excluded. The derived variables were classified into four groups: Group 1 - days of delirium, Group 2 - delirium episodes; Group 3 - maximum time of consecutive positive delirium; Group 4 - delirium density (days of consecutive positive delirium/days of delirium). These variables were compared with the outcome - mortality - of patients with positive delirium during the ICU stay. SPSS 21 for Windows was used for statistical analyses.

## Results

Forty-five patients were analyzed, 16 of whom presented delirium. The mortality of delirium patients was 60%. Group 1: 1 day with delirium was associated with mortality of 60% compared with 50% with 2 days or more. Group 2: patients who had just one episode had 67% mortality compared with 50% mortality if they have two or more episodes. Group 3: mortality with a density ≥0.5 was 57% versus 50% mortality in the subgroup <0.5. Finally, Group 4: comparing the patients who died with the survival patients, we found 1.75 days of consecutive positive evaluation in the patients who died and 4 days in those who survived. None of the differences between these results for any of the four groups were statistically significant.

## Conclusion

Owing to the small population analyzed we could not conclude which was the best variable to predict delirium outcomes. None of the variables analyzed affected outcome when compared with just one positive evaluation during the ICU stay. New studies with a larger population are needed to identify the best variable.

